# Supplementary figures and images for: The Role of AKR1B10 in Lung Cancer Malignancy Induced by Sublethal Doses of Chemotherapeutic Drugs
Source: Cancers (Basel). 2024 Jul 1;16(13):2428. doi: 10.3390/cancers16132428 (PMC11240762; doi:10.3390/cancers16132428)

**Figure 4B**

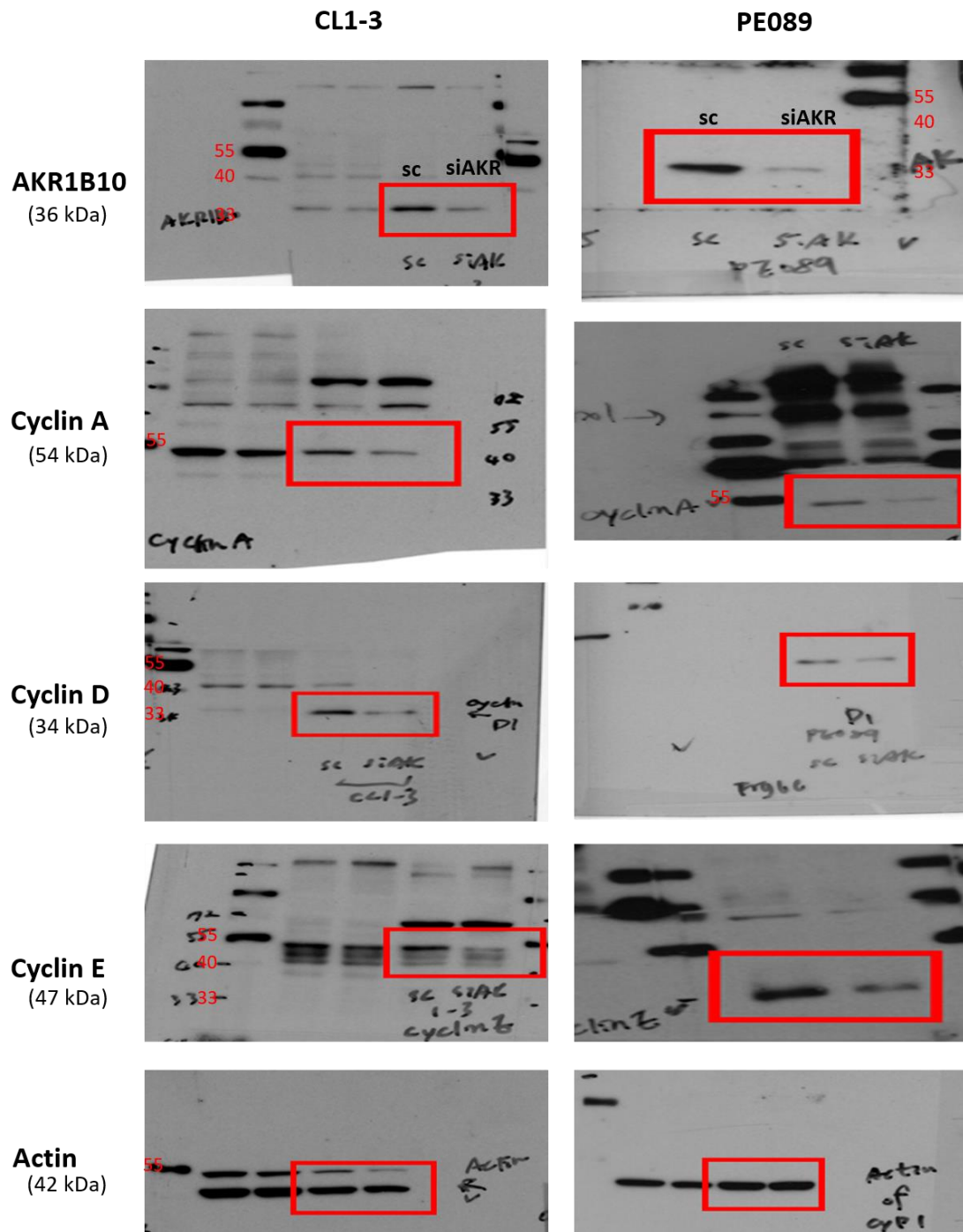

Figure 6D

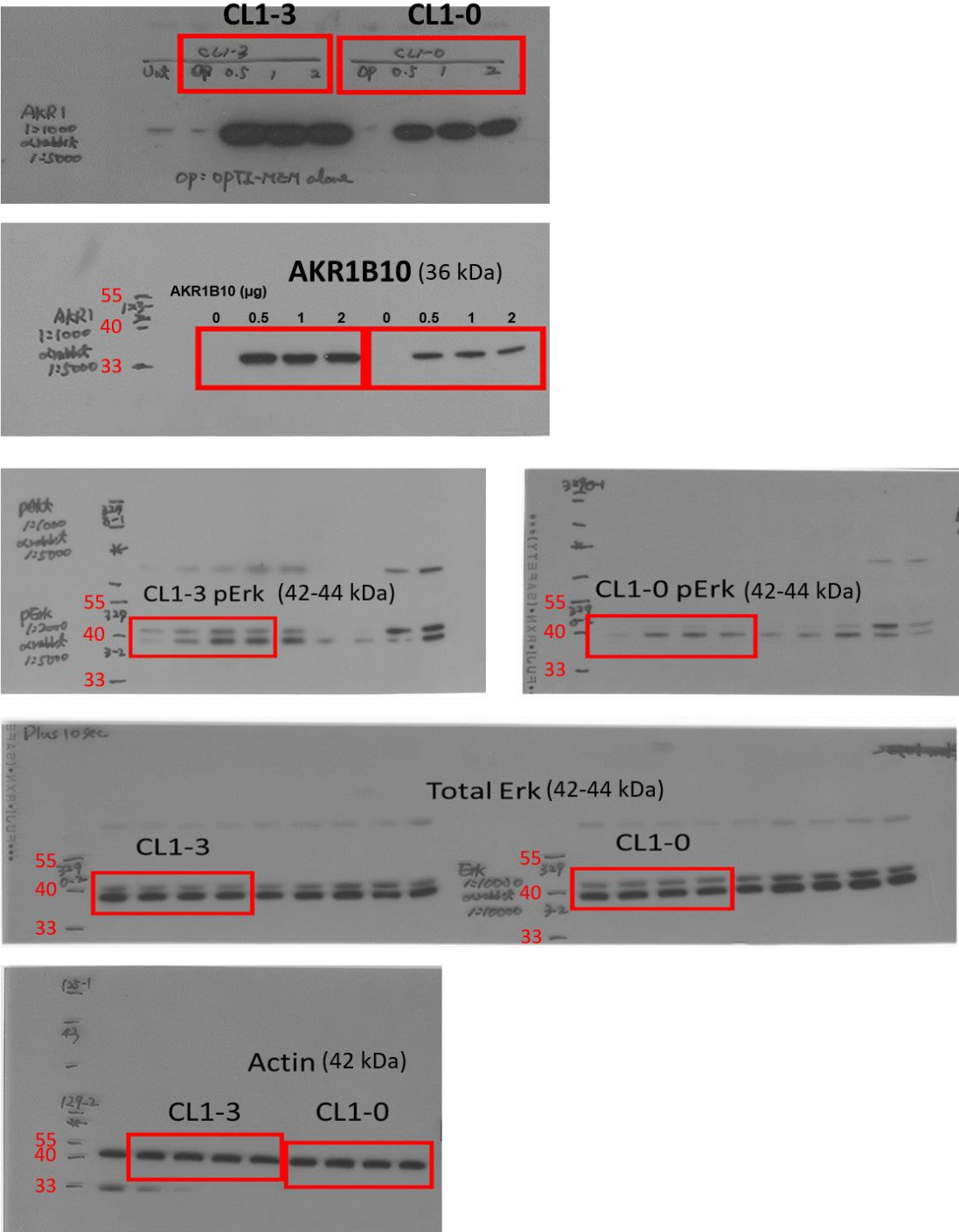

Supplement: Supplementary file 1 [file cancers-16-02428-s001.zip › cancers-3059189-supplementary.pdf]
